# Normal Limit for Serum Alanine Aminotransferase Level and Distribution of Metabolic Factors in Old Population of Kalaleh, Iran

**DOI:** 10.5812/hepatmon.10640

**Published:** 2013-10-20

**Authors:** Ali Kabir, Akram Pourshams, Masoud Khoshnia, Fatemeh Malekzadeh

**Affiliations:** 1 Department of Epidemiology, Faculty of Public Health, Shahid Beheshti University of Medical Sciences, Tehran, IR Iran; 2 Center for Educational Research in Medical Sciences, Tehran University of Medical Sciences, Tehran, IR Iran; 3 Digestive Disease Research Center, Shariati Hospital, Tehran University of Medical Sciences, Tehran, IR Iran; 4 Golestan Research Center of Gastroenterology and Hepatology, Golestan University of Medical Sciences, Gorgan, IR Iran

**Keywords:** Alanine Aminotransferase, Age Groups, Gender

## Abstract

**Backgrounds:**

Normal or elevated values of serum alanine aminotransferase level (ALT) vary in different studies mostly related to characteristics of reference population including age, gender, body mass index, nonalcoholic fatty liver disease (NAFLD), and metabolic syndrome prevalence.

**Objectives:**

To measure upper normal limit (UNL) for serum ALT in an apparently healthy Iranian old population (which we had not sufficient data before this study), and its modulating factors.

**Patients and Methods:**

All inhabitants (> 50 years old) of Kalaleh, Golestan, Iran (N = 1986) were invited to the study. ALT measurements were performed for all subjects using the same laboratory method. Upper limit of normal (ULN) ALT was calculated based on its 95th percentile in normal weight subjects. Modulating factors of ALT were determined by multivariate analysis.

**Results:**

A total of 1309 subjects, with the mean age of 61.5 ± 7.5 years were included. UNL of ALT was 18.8 U/L and 21.4 U/L in women and men, respectively. Based on univariate analysis, waist circumference (r = 0.124, P = 0.01), body mass index (r = 0.118, P = 0.01), triglyceride (r = 0.143, P = 0.01), and having metabolic syndrome (OR = 2.04) modulate ALT levels in men. Also triglyceride (r = 0.119, P = 0.01) modulates ALT levels in women.

**Conclusions:**

The calculated level for UNL of ALT is considerably far lower than previous accepted value. Age, gender, ethnicity, and metabolic factors should be accounted in future studies to determine normal ALT level.

## 1. Background

Serum alanine aminotransferase (ALT) level is a valid and sensitive indicator of liver-cell damage (1, 2). ALT is an important parameter for screening, diagnosis and follow-up of liver diseases (1, 2). Upper Limit of Normal (ULN) of serum ALT level should be able to differentiate between a healthy person and asymptomatic patient with a liver disease. ULN of ALT has been set at 40 U/L since 1950s, when serum ALT levels were used as a surrogate marker for non-A, non-B hepatitis among blood donors before identifying hepatitis C virus (HCV)(3). At that time, it was assumed that reference populations for measuring ULN of ALT included many asymptomatic patients with nonalcoholic fatty liver disease (NAFLD), so calculating ALT levels has not been performed in healthy populations. Nowadays, NAFLD is the most common cause of asymptomatic ALT elevation (4). ULN of ALT should be able to be used for screening NAFLD, too. ULN of ALT varies in different studies mostly related to characteristics of reference population including age, gender, body mass index, NAFLD, and metabolic syndrome prevalence (4, 5).

## 2. Objectives

The aim of this study was to investigate ULN for serum ALT and its modulating factors in an Iranian old population. This study also focuses on metabolic syndrome (MS) and its component and its association with ALT in this healthy appearing population.

## 3. Patients and Methods

The institutional review board (IRB) of the Digestive Disease Research Center (DDRC) of Tehran University of Medical Sciences reviewed and approved the study protocol and the informed consent forms. The DDRC Medical Ethics Committee has been registered in the Office for Human Research Protection (The US Department of Health and Human Services) under the registration number of IRB1641. Between July 2006 and January 2007, all men aged 50 to 79 and women aged 55 to 79 years old resident in Kalaleh, Golestan; Northern Iran, (N = 1986) were invited to the study. After explanation to the participants about the aim and method of the study a written informed consent was obtained. Subjects were interviewed by two trained general practitioners. Information about demographic characteristics, medication history especially during the past 6 months, alcohol consumption (the number and type of drinks per day) and cigarette and opium usage were obtained. In addition, body mass index (BMI) and two seated blood pressures, 1 minute apart after 5 minutes rest, were determined for all. BMI was calculated as weight (kg) divided by height squared (m^2^) and categorized according to the classification of the National Heart, Lung and Blood Institute of the USA as follows: underweight (< 18.5 kg/m^2^), normal weight (18.5–24.9 kg/m^2^), overweight (25–29.9 kg/m^2^), and obese (≥ 30 kg/m^2^) (6).

The subjects who had any diagnosed liver disease, viral hepatitis, renal failure, any kind of cancer, acute infection, and who were taking antibiotic, insulin, corticosteroid and also alcohol drinkers (subjects with an intake of more than 20g alcohol per day) were excluded.

Fasting (8-10 hours) blood venous samples were taken and centrifuged within 30 minutes of collection. All biochemistry tests were performed at the Endocrine and Metabolism Research Center, Tehran University of Medical Sciences, Tehran, Iran. Analyses of serum ALT levels were performed by using the Hitachi 704 auto analyzer, (Tokyo, Japan) with Pars Azmoon reagents kit (Tehran, Iran). Cholesterol, high density lipoprotein (HDL), low density lipoprotein (LDL), and triglyceride (TG) were measured using an enzymatic-photometric analyzer (Pars Azmoon Co., Iran).

The ULN for aspartate aminotransferase (AST) and ALT introduced by manufacturer was 40 U/L for both men and women. Metabolic syndrome (MS) definition was made according to the new International Diabetes Federation (IDF) definition (Central obesity plus any two of the following factors: raised TG level, reduced HDL cholesterol, raised blood pressure, and raised fasting blood sugar (FBS)) (7). Raised levels of fasting blood sugar and TG were considered ≥ 100 and 150 mg/dL, respectively. HDL level was abnormal if it was lower than 40 in men or 50 mg/dL in women (7). Central obesity was considered if waist circumference (WC) was ≥ 94 cm in men and ≥ 80 cm women according to the IDF definition for Europeans. We also used Iranian cutoff of WC to identify metabolic syndrome in our analysis including Delavari A et al. (8), Azizi F et al. (9), and Gharipour M et al. (10) results. In Delavari A et al. study optimal cutoff point of WC to predict metabolic syndrome as defined by the IDF was 89 cm for men and 91 cm for women (8). Azizi F et al, found waist ≥ 95 cm in both genders for identifying metabolic syndrome (9). The cut points for WC were nearly equal in men and women, 90.3 cm versus 90.0 cm, respectively, in Gharipour M et al. study (10).

Statistical analysis was performed using SPSS, version 16, Software (SPSS, Inc., Chicago, IL, USA) and STATA 9.1 software (STATA Corp. LP). Normal distribution of quantitative variables was checked by both statistical tests (Kolmogorov-Smirnov and Shapiro test) and graphs (Q-Q plot and histogram). Continuous variables were analyzed with t-test and one way ANOVA and categorical variables with chi-square. Mann-Whitney test was used for comparing medians of ALT in both genders by assuming that the ALT distributions in both genders have the same pattern. Pearson correlation coefficient was used to show the strength of any significant association between quantitative variables. We used partial correlation in which the effect of confounder variables would be considered fix, to assess the correlation between quantitative variables. Odds ratio (OR) and its 95% confidence interval (95% CI) were also calculated to assess the strength of the differences for dichotomized variables. Considering ALT as both qualitative and quantitative variable, we approached its association with other measured variables in the study. Regression models were used for deleting the confounder effects of other variables. Logistic regression analysis using the forward Wald method was performed to determine the adjusted OR of the most important factors for abnormal (elevated) ALT.

We included variables with a p-value of 0.2 or less in their bivariate analysis with ALT. Variables that could significantly change the chi-square of the model remained in the final model. A two-sided P value less than 0.05 was considered statistically significant.

## 4. Results

From 1986 invited subjects, 1733 persons participated in the study (response rate = 87.3%). Among them, 4 cases did not consent, and 420 persons were not eligible. Finally a total of 1309 subjects were entered the study, 688 (52.6%) were male. ULN of ALT in males and females were 21.4 and 18.8 U/L, respectively which were statistically different (P < 0.001). Table 1 shows other demographic data and ALT levels of the participants. 

**Table 1.** Demographic Data and ALT Levels

	Male (N = 688)	Female (N = 621)	P value	Total (N = 1309)
**Age, y, mean ± SD**	61.5 ± 7.9	61.4 ± 7.9	0.84	61.5 ± 7.5
**ALT ^**a**^ , mean ± SD, U/L**	20.5 ± 11.9	17.9 ± 11.2	< 0.001	19.3 ± 11.6
**Median of ALT, U/L**	18	15	< 0.001	16
**95% CI of ALT, U/L**	19.6-21.4	17-18.8	< 0.001	19.9
**ALT > 40, U/L (prevalence, %)**	4.9	3.5	< 0.001	4
**ALT > Current ULN (Prevalence, %)**	35.8	36.8	0.707	38
**WC ^**a**^ , mean ± SD, cm**	92.7 ± 11.7	93.9 ± 12.7	0.093	93.3 ± 12.2
**BMI ^**a**^ , mean ± SD, kg/m**	25.9 ± 4.2	28.2 ± 5.3	< 0.001	27 ± 4.9
**BMI ≥ 30 kg/m2, %**	17.4	33.5	< 0.001	25.1
**History of HTN** ^**a**^ **, %**	18.6	31.6	< 0.001	24.8
**History of diabetes mellitus, %**	10.9	16	0.007	13.3
**Blood pressure≥ 130/85, %**	61.8	66.3	0.890	63.9
**FBS ^**a**^ ≥ 100 mg/dl, %**	40.9	40.5	0.897	38.7
**TG ^**a**^ ≥ 150 mg/dl, %**	37.9	44.5	0.018	39.1
**HDL ^**a******^ < 40 in men, HDL < 50 mg/dl in females, %**	43.7	53.1	0.001	45.8
**MS ^**a**^ (IDF definition and WC for European), %**	23	37.2	< 0.001	29.9
**MS (IDF definition and WC ≥ 95 cm), %**	21.5	20.8	0.846	21.1

^a^ Abbreviations: ALT, alanine aminotransferase; BMI, body mass index; CI, confidence interval; FBS, fasting blood sugar; HDL, high density lipoprotein; HTN, hypertension; IDF, International Diabetes Federation; MS, metabolic syndrome; TG, triglyceride; WC, waist circumference

ALT levels > 40 U/L was observed in 53 subjects (4%), far lower than the prevalence of obesity and metabolic syndrome (Table 1). When we used our calculated normal cutoff values for men and women (21.4 and 18.8 U/L, respectively) the prevalence of elevated ALT increased to 38% which is absolutely more comparable to the prevalences of obesity (25%) and MS (21-29.9%) in the population. 

Mean ALT level was significantly higher in men with MS (IDF definition and WC ≥ 95Cm) than men without MS (23.6 ± 12.5 vs. 19.6 ± 11.6, P < 0.001). Abnormal ALT (according to the definition made by the present study) was also associated with MS in men (IDF definition and WC ≥ 95Cm) [OR (95% CI): 2.04 (1.41, 2.96), P < 0.001] but not in women.

There was no difference between the mean ALT levels in females with and without MS (IDF definition and WC ≥ 95Cm) (17.95 ± 8.9 vs. 17.86 ± 11.8, P=0.936).

Elevated WC and TG, low HDL, obesity, and MS (IDF definition and European WC) were more prevalent in women than men (Table 1). The prevalence of blood pressure ≥ 130/85 mmHg and FBS≥ 100 mg/dL were 64% and 39% respectively, without significant difference in men and women. Only about 30% and 25% of women and men respectively were aware of having hypertension or DM. The prevalence of MS was equal in men and women based on the newly defined Iranian cutoff (WC ≥ 95 cm). 

Serum ALT level was correlated weakly with BMI, WC, and TG level in men but only with TG level in women (Table 2). 

**Table 2.** Correlation Between Metabolic Syndrome Parameters and Serum ALT Level ^a ^

	BMI ^b^	WC ^b^	SBP ^b^	DBP ^b^	TG ^b^	HDL ^b^	FBS ^b^	ALT ^b^
**BMI**	-	0.847	0.217	0.386	0.302	-0.203	0.123	0.118
**WC**	0.815	-	0.215	0.373	0.312	0.236	0.163	0.124
**SBP**	NS	NS	-	0.651	0.121	NS	0.081^c^	NS
**DBP**	0.237	0.27	0.681	-	0.2	-0.078^c^	NS	0.079^ c^
**TG**	0.095^c^	0.122	0.86^c^	NS	-	-0.442	0.221	0.143
**HDL**	NS	NS	NS	NS	-0.483	-	-0.1	NS
**FBS**	NS	NS	0.1 ^c^	-0.087^c^	0.293	-0.153	-	NS
**ALT**	NS	NS	-0.084 ^c^	NS	0.119	NS	NS	-

^a^ Numbers are Pearson correlation coefficients for men in the upper right of the table, for women in the lower left of the table

^b^ Abbreviations: ALT, alanine aminotransferase; BMI, body mass index; DBP, diastolic blood pressure; FBS, fasting blood sugar; HDL, high density lipoprotein; NS, non-significant; SBP, systolic blood pressure; TG, triglyceride; WC, waist circumference

^c^All correlations were significant at the level of P = 0.01, except for these at the level of P = 0.05

Prevalence of central obesity and hypertriglyceridemia increased according to the increase in serum ALT level in men but not in women (Table 3). 

**Table 3.** Percentage of Abnormality in Metabolic Syndrome Criteria by Serum ALT Level

Metabolic abnormality	ALT^a^, U/L					P value for trend
	< 20	20-29.9	30-39.9	40-49.9	≥ 50	
**Men**						
WC ^a^> 89 ^b^	57.4	71.2	68.7	70	52.9	0.015
WC > 90.3 ^c^	52.9	65.2	65.7	70	47.1	0.021
WC ≥ 94 cm ^d^	42.9	54.3	55.2	70	41.2	0.014
WC ≥ 95 ^e^	39.4	49.5	49.3	70	35.3	0.016
SBP ^a^ ≥ 130 or DBP ^a^ ≥ 85	60.7	64.2	61.2	55	64.7	0.896
FBS ^a^≥ 100	38.2	42.2	47.8	55	41.2	0.377
TG ^a^ ≥ 150	31	43.3	49.3	70	52.9	< 0.001
HDL ^a^< 40	59.4	51.9	58.2	40	47.1	0.208
**Women**						
WC > 91 cm ^b^	57	57.9	53.5	60	45.5	0.929
WC> 90 ^c^	59.1	64.9	58.1	70	45.5	0.603
WC ≥ 80 ^d^	85.2	88.6	90.7	90	72.7	0.497
WC ≥ 95 ^e^	47.2	47.4	48.8	40	27.3	0.740
SBP ≥ 130 or DBP ≥ 85	68	65	68.2	60	72.7	0.956
FBS ≥ 100	37.4	47.1	51.2	20	54.5	0.08
TG ≥ 150	41.9	46.2	63.6	30	54.5	0.06
HDL < 50	51.7	53.8	61.4	50	63.6	0.722

^a^ Abbreviations: ALT, alanine aminotransferase; DBP, diastolic blood pressure; FBS, fasting blood sugar; HDL, high density lipoprotein; SBP, systolic blood pressure; TG, triglyceride; WC, waist circumference

^b^ according to the reference number (8)

^c^according to the reference number (10)

^d^ according tothe reference number (7)

^e^according to the reference number (9)

Table 4 shows factors modulating ALT levels in univariate analysis. By increasing age and HDL, ALT decreases. In contrast, increase in blood pressure, WC, BMI, TG, and male gender have direct effect on ALT increment. FBS did not modulate ALT level. 

**Table 4.** Factors Modulating Serum ALT Levels

	Odds ratio	95% confidence interval (lower, upper)
**Blood pressure ( ≥ 135/85), mmHg**	2	(1.5, 2.7)
**Age, y**	0.97	(0.95, 0.98)
**Male gender**	1.6	(1.3, 2)
**WC ** ^**a**^	1.02	(1.01, 1.03)
**BMI ** ^**a**^	1.03	(1.003, 1.05)
**HDL ** ^**a**^	0.98	(0.97, 0.99)
**TG** ^** a**^ ** (per 10 mg/dl)**	1.02	(1.01, 1.04)

^a^ Abbreviations: BMI, body mass index; HDL, high density lipoprotein; TG, triglyceride; WC, waist circumference

In multivariate logistic regression analysis, only TG level was associated weakly with ALT level [OR (95% CI): 1.05 (1.001, 1.09)]. There was a correlation between BMI and ALT level in men (r = 0.118, P = 0.002) but not women (Table 5). 

**Table 5.** UNL for ALT in Normal Weight, Overweight and Obese Patient

BMI ^a^, kg/m^2^	Female UNL ^a^ of ALT ^a^, U/L	Male UNL of ALT, U/L
**< 25**	19.1	20.7
**25-29.9**	19.5	22.6
**≥ 30**	19.8	23.8

^a^ Abbreviations: ALT, alanine aminotransferase; BMI, body mass index; ULN, upper limit of normal

## 5. Discussion

WC, BMI, TG and having MS modulate ALT levels in men. TG modulates ALT in women. For almost 50 years, it was accepted that UNL of ALT is about 40 U/L (3). Several studies have recently challenged the cutoff for normal ALT level (11, 12). The first Iranian study for determining UNL of ALT in adult was performed in 2002 among blood donors (10). A total of 1959 apparently healthy blood donors with the mean age of 37.4 years were recruited for the study in Tehran Blood Donation Center. UNL of ALT was calculated 40 U/L for men and 34 U/L for women among blood donors with normal weight (BMI< 25 Kg/m^2^). Serum ALT levels were independently associated with BMI and sex, and association of ALT was more prominent in male than female (13). The study had two limitations. The population of study was not nationally representative of Iran, and there was no laboratory data regarding associated factors with serum ALT levels.

Second Iranian study for determining UNL of ALT for adult was performed in 2006 among inhabitants in north-eastern Iran (14). Rural and urban dwellers both were included in the study. UNL of ALT was 37.5 U/L and 36 U/L in male and female respectively in normal weight participants. Male gender, high BMI and having history of diabetes mellitus were independent risk factors for ALT elevation. Same to the study in Iranian blood donors, lack of general ability and laboratory data regarding associated factors were limitations of the study.

In the current study, we recruited most healthy inhabitants in Kalaleh, Golestan, who were older than 50 years, and were candidate to recruit in a clinical trial for using medication for primary prevention of ischemic heart disease (15).

The strong points of the current study are as follows; the population had a very detailed history of any chronic diseases and relevant laboratory data. Although the current reference population for determining UNL of ALT had relevant laboratory data, but there was a limitation in age groups. So, we were not able to identify the influence of age on serum ALT level and also other factors which are probably present in younger population.

In the study of Elinav E et al. a significant association was found between age and ALT levels, made an inverted U like curve with a peak of serum ALT level at 40-55 years (16). The Mean serum ALT level was 19 ± 13 U/L in < 40 years old, 25 ± 19 U/L in 40-55 years old, 22 ± 10 U/L in 56-72 years old, 17 ± 9 U/L in 73-83 years old, and 13 ± 5 U/L in 83-100 years old (P < 0.0001) (13). In Iranian blood donors the peak of serum ALT levels was at 40 - 50 years of age too (13). We concluded that the population age was the main reason for calculating far lower level of normal ALT in our population comparing to the two previous Iranian investigations. So, it is important to consider age for interpretation of an ALT level in different ages as well as gender.

The influence of BMI on the UNL of ALT has been shown in the two previous Iranian studies (13, 14). In the current study, the effect of BMI on ALT activity was observed only in men. Leclercq I et al. studied the influence of age, BMI and sex on ALT levels among 9420 blood donors (17). Although Leclercq I et al. found a correlation between BMI and ALT level, but consistent influence of BMI on ALT level was observed only in women. In men, raising in BMI increased ALT activity only up to the fifth decade and after that no influence was seen any more (17). In Hsieh MH et al. study, 11411 Taiwanese adults were enrolled (18). Hsieh MH et al. found that WC, BMI, TG, and blood sugar are important risk factors for elevated ALT, and WC might be a better indicator of risk of abnormal liver function than BMI (18). In our study, BMI and WC were associated with higher level of ALT only in men. The association between ALT levels and WC in men was observed even in normal and near normal range of ALT levels (Table 3). The cutoff of WC that predicts metabolic syndrome in Iranian women is higher than calculated cutoff for European (7) and east-Asian women (18). The predictive cutoff of ALT for Iranian women is almost the same to the Iranian men (8- 10), and is not comparable with European or Asian countries (7, 18). Theses suggest that gender and ethnic differences in amount of visceral fat may play a role in the association between WC and ALT levels. 

We found that blood pressure modulates ALT level in univariate analysis significantly; but, not in multivariate analysis that is comparable with Hsieh MH et al. study results (18). We did not find an association between elevated FBS and serum ALT levels. Saligram S et al. found that ALT level is associated with BMI, hypertriglyceridemia and low HDL but not with hemoglobin A1C and glycemic control (19). We found an association between ALT level and MS in men but not women. Most studies are reporting an association between serum ALT level and MS in various populations including elderly men (20-22).

There is no convincing data to answer whether MS or any individual component of MS is an important predictor of liver damage, indicated by an elevated ALT level in different age, sex and ethnicity. In a study from Iran, authors found high blood pressure in both genders, high WC in men and high FBS in women were independent predictors of cardiovascular disease and adding other MS parameters did not yield any improvement in model fitness for predicting cardiovascular disease (23). In our study, hypertriglyceridemia modulated ALT levels in both genders, but WC, BMI and MS were associated with ALT levels only in men .There was no association between high blood pressure and high FBS with ALT in both genders. It seems that the influence of individual parameters of MS for predicting an ALT elevation and a liver injury is different in men and women, which needs to be evaluated in a series of prospective studies. In the current study, 23% and 37.2% of men and women had MS based on the IDF definition which is comparable with the first Iranian national survey of MS which conducted in 2007 on 3024 subjects. The authors found that 37.4% of population has MS based on IDF definition. The prevalence of MS was higher in women and in the 55 -64 years old age group (8).

There is conflicting data regarding the influence of ethnicity on ALT level. Schwimmer JB et al. studied the influence of ethnicity, race and gender on ALT level in obese adolescents in a national school based survey in the USA (24).

After controlling for BMI and sex, Hispanic had significantly more ALT levels than black and white. Influence of BMI, gender and ethnicity on ALT level was studied by Bilal M et al. among students of an army medical college in Pakistan (25). The authors found that ALT levels strongly correlated with BMI and gender but not with ethnicity.

We evaluated opium usage and its duration based on a standard questionnaire and our previous study using urine codeine or morphine as the gold standard method for detecting the use of opium, showed that self-report of this variable had a sensitivity of 0.93 and a specificity of 0.89. Its reliability was also high (26). So, the recall bias even for the duration of using opium was not considerable.

In summary, our current calculated UNL of ALT (21 U/L and 19 U/L in male and female, respectively) is far lower than the upper normal limit for ALT level which had been determined by laboratory manufacture. We assumed that it is due to the age category of our population. Using current UNL of ALT increases the detection rate for serum ALT elevation from 4% to 38%, which is more expected considering the prevalence of MS and its parameters which modulate ALT level. MS and its components are very prevalent in our population. There is a lack of data regarding modulating factors of ALT levels in different ethnicity, gender and age groups. Future prospective studies for calculating UNL of ALT should take into account age, gender, ethnicity and metabolic factors.
